# Supplementary material for: Research on customer churn prediction and model interpretability analysis
Source: PLoS One. 2023 Dec 8;18(12):e0289724. doi: 10.1371/journal.pone.0289724 (PMC10707658; doi:10.1371/journal.pone.0289724)
Supplement: Attachment — Submitted filename: Response to Reviewers.docx [file pone.0289724.s002.docx]

Dear Reviewers,

Thank you very much for your time involved in reviewing the manuscript and your very encouraging comments on the merits.

We also appreciate your clear and detailed feedback and hope that the explanation has fully addressed all of your concerns. In the remainder of this letter, we discuss each of your comments individually along with our corresponding responses.

To facilitate this discussion, we first retype your comments in italic font and then present our responses to the comments.

**Reviewer #3:**

**Comment 1:**

*author has worked on various sections to improve the paper. There are improvement from the previous attempt. However, i would still suggest to improve the discussion section.*

**Author response 1:** We appreciate your valuable comments and we have modified and adjusted the Discussion section accordingly. We have added a discussion section describing the results of the experiment as follows.

Table 5 shows the results of processing the unbalanced data. In the experiment, the data was balanced using SMOTE, ADASYN, and SMOTEENN. From the results of the experiments, it is clear that the model using the balanced data technique improves the performance of the model in a better way compared to the model that does not perform any manipulation on the data. The SMOTEENN resampling method gives the best performance in the XGBoost model when comparing several methods. The accuracy and recall of the model with the SMOTEENN method reach 96% and 92%, respectively.

Tables 7 and 8 show the results of parameter optimization. Hyperparameter tuning plays an important role in constructing models using ensemble learning approach, and genetic algorithm is used as an improved solution in this study, and the outstanding improvement of genetic algorithm compared to traditional parameter optimization methods can be clearly reflected from the results. After data balancing of XGBoost using SMOTEENN, the parameters of XGBoost are optimized using genetic algorithm, and the final optimized parameters are n_estimators=265, learning_rate=0.097, max_depth=5. From the recorded results, it is clear that the genetic algorithm improves XGBoost better than the grid search with the random search methods better improves the performance of the XGBoost model. From Figure 10, it can be seen that the GA-XGBoost model tuned by genetic algorithm achieves the highest AUC value, which reaches a value of 99.02%. The comparison of the model results in Table 9 shows that the GA-XGBoost model outperforms other machine learning models in every evaluation metric. It is clear that the application of the GA-XGBoost method significantly improves the performance of user churn prediction in the banking industry. **[Line 475-Line 490]**
